# What Makes for Healthy Ageing in the Torres Strait?

**DOI:** 10.1111/ajr.70020

**Published:** 2025-03-07

**Authors:** Chenoa Wapau, Malcolm McDonald, Fintan Thompson, Rachel Quigley, Sarah G. Russell, Betty Sagigi, Gavin Miller, Tania Korinihona, Edward Strivens

**Affiliations:** ^1^ Torres and Cape Hospital and Health Service Queensland Health Brisbane Queensland Australia; ^2^ College of Medicine and Dentistry James Cook University Cairns Queensland Australia; ^3^ Australian Institute of Tropical Health and Medicine James Cook University Cairns Queensland Australia; ^4^ Cairns and Hinterland Hospital and Health Service Queensland Health Cairns Queensland Australia; ^5^ Metro North Health Service Queensland Health Brisbane Queensland Australia

**Keywords:** dementia, First Nations People, healthy ageing, healthy ageing index, strengths based, stroke, Torres Strait

## Abstract

**Objective:**

Many studies focus on impediments to healthy ageing, but few examine factors leading to healthy ageing. Whilst many older First Nations people are ageing well, few studies have examined this issue in First Nations people. This study examined indicators associated with healthy ageing in the Torres Strait region of Queensland, Australia.

**Methods:**

Data from a Torres Strait Dementia Prevalence Study (2015–2018) were used to explore indicators of healthy ageing in 249 participants. A specific Torres Strait Healthy Ageing Index was created, based on 10 indicators from the dataset. One point was assigned for each indicator, with higher scores representing healthier ageing. This Index was then used to assess healthy ageing in a subset of participants aged 70 years and older.

**Results:**

Healthy ageing scores were higher in younger people. However, among 80 people aged ≥ 70 years, many were ageing well according to the healthy ageing index, with 44% scoring 7–8 and 28% scoring 9–10. Age‐adjusted analyses identified that more education, lack of vascular risk factors, good medication prescribing patterns, absence of stroke and geographic location were all associated with a higher healthy ageing index.

**Conclusions:**

Our study suggested that many older First Nations residents of the Torres Strait region were ageing in a healthy way, based on the indicators available. The results showed both predictable and unexpected associations with healthy ageing. Stroke prevention stood out as a key priority. These results highlight the need for prospective population studies to better understand, promote and celebrate healthy ageing in this population.

**Summary**

- What is already known about this topic?
  ○ Research with First Nations Peoples has traditionally taken a deficit discourse, which fails to celebrate the strengths within communities that can be harnessed to promote ageing well.
  ○ This includes measures of Healthy Ageing, based on mainstream indices, which are not always appropriate for Indigenous populations.
- What this paper adds?
  ○ A new Torres Strait‐focused healthy ageing index has been developed.
  ○ This new healthy ageing index shows that many older First Nations Australians in the Torres Strait are ageing well, although chronic disease continues to be a concern.
  ○ Chronic disease prevention measures are a key priority for health services, but other factors such as education and judicious prescribing also influence ageing well and also require appropriate interventions.

## 1 Introduction

Healthy ageing is an honour enjoyed by too few people. To grow old and remain active is a privilege, whether hard‐earned or by sheer luck. The World Health Organisation defines healthy ageing as ‘the process of developing and maintaining the functional ability that enables wellbeing in older age’ [1]. Functional ability in this context refers to health‐related factors that allow people to have their basic needs met, to be able to learn, develop and make decisions, maintain mobility, build and sustain relationships and contribute to society. Whilst older adults may be living longer, particularly in developed countries, such increased longevity does not always equate to extended periods of good health [2]. For this reason, it is important that healthy ageing is defined and operationalised so that it can be objectively measured within different populations [3].

The last 20 years have seen the publication of numerous healthy ageing indices and scores. A recent review by Michel et al. [4], identified indices that assess healthy ageing at a population level. These include the Global Age Watch Index [5], which is used worldwide, and the Active Ageing Index, which is used more frequently within the European Union [6]. A commonly cited index that is used to characterise ageing for individuals is the Healthy Ageing Index, developed in the United States by Sanders et al. [7, 8] This index covers systolic blood pressure, nonfasting plasma glucose levels, global cognitive functioning, plasma creatinine levels and lung functioning. There have been modifications to these indices [9]. For example, in 2018, the United Nations Economic Commission for Europe published the above‐mentioned Active Ageing Index with an assessment tool that covers 22 indicators in four domains [10]. The indicators are functionally based, and users have produced large, published datasets, mainly from high‐income countries. It should be noted that all these indices share some limitations; none have been formally validated [11], and none appear to have been developed for ethnic, diverse or First Nations populations.

The people of the Torres Strait include those living on the islands between Cape York and Papua New Guinea and the mainland communities of the Northern Peninsula Area [NPA]. The Torres Strait and NPA region comprises 18 island communities and five mainland communities totalling more than 8300 people who identify as First Nations People [12]. Torres Strait Islander people are one of two culturally distinct First Nations populations in Australia. The population is remarkably diverse but is strongly united by culture, family tradition and religion. Daily living decisions are often dictated by the environment, including unpredictable weather, unruly seas and unforgiving winds. Transport and communications can be precarious. Compared to the rest of the country, infrastructure resources are limited. As with other First Nations populations, the negative impacts of colonisation continue to drive health disparities within the region. The population endures relatively poor health outcomes when compared to the overall Australian population [13]; with rates of type 2 diabetes mellitus being of particular concern [14] and conditions of older age, such as dementia, becoming increasingly important [15]. Despite the high rates of chronic disease noted in the region, many older Torres Strait Islander people are ageing well.

One criticism of healthy ageing indices is that they often take a deficit discourse by examining barriers to ageing well [16]. This approach has been shown to negatively impact health outcomes, particularly within First Nations Peoples, where deficit‐based approaches have historically been used [16]. First Nation Australians are some of the most over‐researched people on the planet [17, 18]. There are local concerns that much of this research also takes a deficits‐based approach, describing what is wrong rather than finding evidence‐based solutions [17, 19]. This has been a particular issue in the Torres Strait, where there has been a focus on describing the poor health indicators of the local people and identifying risk factors [16]. An alternative approach is to identify the healthy population and explore the factors that keep these people healthy, celebrating and acknowledging their strengths. Of these cultural determinants of health, connection to culture, community, family, Country and place have been shown to promote positive health outcomes and are likely to support ageing well [20]. To date, there has been no known index for assessing healthy ageing in older First Nations Peoples in the Torres Strait.

### 1.1 Context of the Research

A previous dementia prevalence study in the Torres Strait has identified risk and protective factors for dementia in the First Nations population [21]. As the data were collected and analysed, it was also noted that some participants were ageing particularly well. There was a cohort of people 70 years and older who were both healthy and highly functional in terms of being independent with activities of daily living. This prompted the question as to why some people in this cohort aged so well compared to the rest of the participants. There was already an available database of health indices used for the dementia study, which was interrogated further to establish indices of healthy ageing in this population. If there were clear findings, they could potentially point to future preventative health measures and health promotion approaches in the Torres Strait and elsewhere.

The first aim of this retrospective study was to create a functionally based healthy ageing index, tailored to this population. This index could then be used to interrogate the available health indices database. The second aim was to identify index scores that accompanied healthy ageing overall, including among those in the cohort aged 70 years and older. It was hypothesised that there would be clearly identifiable associations between index scores and known health risk factors in older people.

## 2 Methods

The data used for this study were obtained from a Dementia Prevalence Survey (DPS, 2015–2018) previously conducted in the Torres Strait and Northern Peninsula Area (NPA) of Far North Queensland, Australia. The details, including methods and ethics approvals, have already been published [21] and are summarised here.

The DPS was a cross‐sectional survey conducted across 18 islands and in five mainland communities in the Torres Strait and NPA [21]. The study aimed to determine the prevalence of dementia across the region. The development and implementation of the DPS were codesigned and implemented with local health workers, staff and community members. This was overseen by an Indigenous reference group (Knowledge Circle). Recruitment was limited to First Nations residents aged 40 years and over. Participants were recruited by health staff who directly approached clients of the health service and community members. The research team used the Kimberley Indigenous Cognitive Assessment tool (KICA) to collect data on self‐reported social, demographic, medical, cognitive and psychological status [22]. The study also included several self‐reported assessments of function, including falls (Elderly Fall Screening Test, EFT), pain (Brief Pain Inventory, BPI), continence (Modified International Consultation on Continence Questionnaire, ICIQ) and depression (Adapted Patient Health Questionnaire, PHQ‐9). Full details of these measures have been published elsewhere [21]. Participants also underwent a medical assessment by a geriatrician who provided diagnoses of dementia where applicable and independently assessed falls (FROP‐COM Community Fall Risk Screen), hearing and mood. Other demographic variables collected during the DPS included age, gender and years of formal education. Location of residence was defined as living in an ‘inner area’ (i.e., on the mainland, or the central administrative island–Waiben/Thursday Island, or one of the immediate neighbouring islands) and ‘outer area’ (i.e., on one of the outer islands located further away). The number of vascular risk factors was measured as a continuous variable and defined as the cumulative presence of smoking (past and current) and individual vascular risk factors (e.g., diabetes, hypertension, heart disease).

Participants' medication lists were reviewed to identify suboptimal prescribing habits. This included polypharmacy (prescription of ≥ 5 medications), overprescribing (prescribed medication not required for a condition), underprescribing (medications recommended per clinical guidelines not prescribed) and anticholinergic cognitive burden using the Anti‐Cholinergic Burden (*ACB*) *Scale* (prescribing of any medication with an anticholinergic effect) [23]. A ‘sub‐optimal’ prescribing flag was derived and defined as the presence of any of these prescribing types. The criteria and guidelines used included the Beers criteria [24] and the STOPP/START criteria [25].

### 2.1 Healthy Ageing Index

Feedback on the results of the DPS obtained from both community members and the Knowledge Circle was to shift from a deficit‐risk narrative to a strengths‐based approach of valuing healthy ageing. Hence, the research aim to develop a Torres Strait Healthy Ageing Index (TSHAI) was proposed. Data obtained in the dementia prevalence study identified 10 indicators used to develop the TSHAI. These 10 indicators broadly aligned with categories from the International Classification of Functioning, Disability and Health (ICF) [26] and included vision, hearing, mobility, falls, continence, pain, depression, cognition, personal activities of daily living (pADLs) and independent activities of daily living (iADLs). The 10 variables were reviewed and approved by members of the Knowledge Circle.

Table 1 outlines how each indicator was defined based on information from the DPS. Participants were assigned 1 point for each indicator of healthy ageing; a total score was obtained by summing all indicators, with higher scores representing better healthy ageing. For example, a person who had ‘yes’ to all measures had an index of 10. Participants who had missing information on one or more indicators were excluded from the final analyses to ensure all healthy ageing index scores were based on complete case analyses (Table S1).

**TABLE 1:** Definitions of measures of healthy ageing used to derive the Torres Strait Health Ageing Index (TSHAI).

Measure	Definition
1. Vision intact	Yes: An answer of ‘Yes’ to the question: ‘Are your eyes good? Can you see everything’ compared to an answer of ‘No’
2. Hearing intact	Yes: The absence of hearing difficulties from Doctor's medical assessment (i.e., Doctors Letter)
3. Mobility intact	Yes: An answer of ‘No’ to the question: ‘Do you have trouble walking?’, compared to an answer of ‘yes’
4. Minimal falls	Yes: Referred to a geriatrician and received a FROPCOM score of ≤ 15, OR did not see geriatrician and had an EFT score ≤ 2, OR reported no falls and did not have an EFT or see geriatrician No: Referred to a geriatrician and received a FROPCOM score of ≥ 16 (i.e., moderate to severe falls), OR did not see geriatrician and had an EFT score ≥ 3, OR reported ‘Yes’ to falls and did not have an EFT or see a geriatrician
5. Continence intact	Yes: ICIQ ≤ 2, OR did not answer ICIQ and reported no continence issues No: ICIQ ≥ 3, OR did not have ICIQ, but reported having continence issues
6. Minimal pain	Yes: Not referred to geriatrician for pain No: Referred to geriatrician for pain, based on Pain Scale (i.e., in pain all the time, for more than 1‐week duration AND pain was greater than 2/5 on targets AND/OR interfering with activities)
7. No depression	Yes: No Depression and/or Anxiety from Doctor's Letter AND PHQ9 score < 9 No: Doctor's Letter reports Depression and/or Anxiety OR PHQ9 score ≥ 9
8. Cognition intact	Yes: Consensus Diagnosis is NOT Cognitive Impairment Not Dementia (CIND) and NOT Dementia No: Consensus Diagnosis is Cognitive Impairment Not Dementia (CIND) OR Dementia
9. pALDs intact	Yes: Independent with dressing and showering No: Dependent on assistance for dressing or showering
10. iADLs intact	Yes: Independent with cooking, cleaning, medication and finances No: Dependence on assistance for cooking, cleaning, medication or finances

Abbreviations: EFT, elderly falls test screening tool; FROPCOM, the falls risk for older people in the community screening tool; iALDs, independent activities of daily living; ICIQ, the international consultation of incontinence screening tool; PHQ‐9, patient health questionnaire; pALD, personal activities of daily living.

### 2.2 Statistical Analysis

**FIGURE 1:**
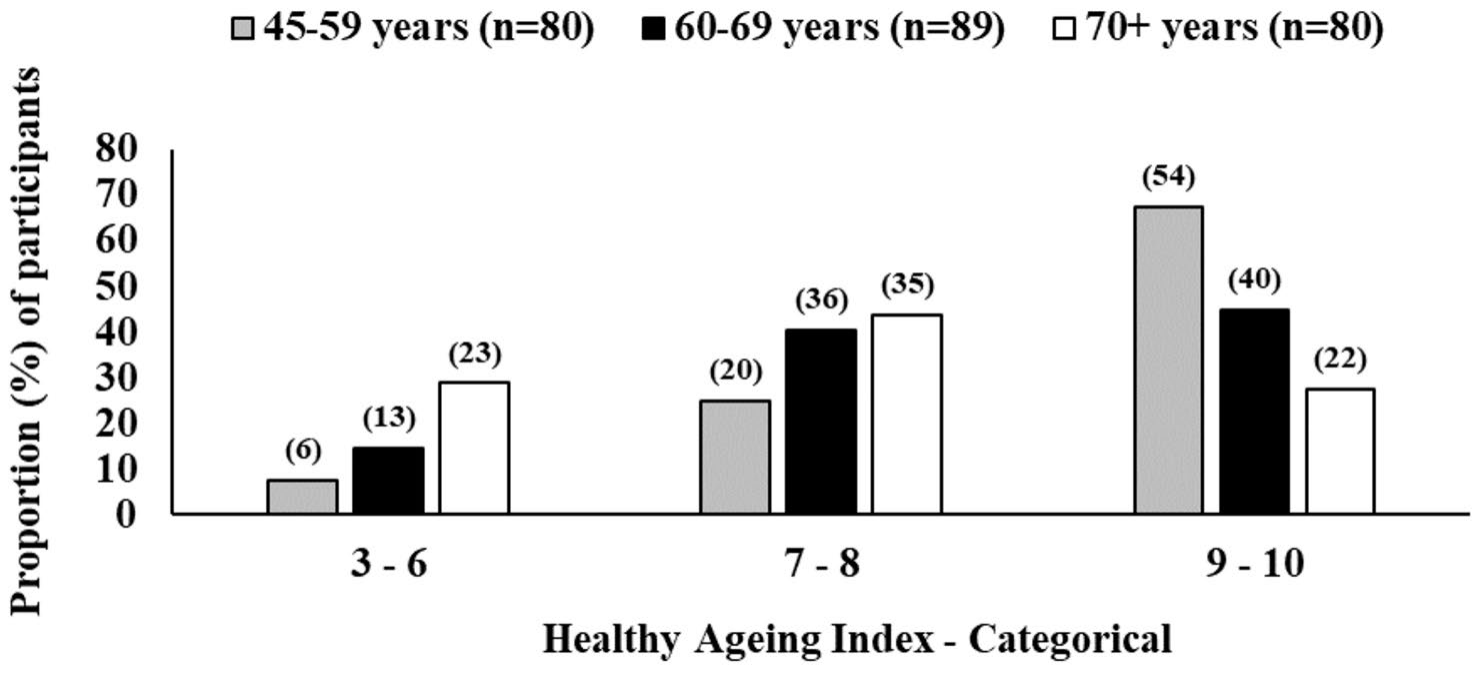
Distribution of the Torres Strait Healthy Ageing Index (TSHAI) in three categories (3–10), by three age groups (45–59, 60–69 and 70+), for 249 participants.

The TSHAI was analysed as a continuous variable and also as a categorical variable grouped into three categories (i.e., 3–6, 7–8 and 9–10) (Figure 1). The associations between the categorical healthy ageing index and demographic, lifestyle and medical information were examined using Chi‐square analyses (Table 2). When data were missing for certain variables, proportions were calculated excluding missing cases. For example, the education level was missing for seven participants, so these participants were excluded from the denominator. The categorical health ageing index was analysed, adjusted for age in ordered probit regression models, using the Stata Version 15 command ‘oprobit’. The TSHAI had small cell sizes in three categories, which may have affected adjusted probit models. Probit models are also not intuitive to interpret, as the values taken on by the dependent variable are arbitrary except when they correspond to higher outcomes. Given these limitations, the TSHAI was also examined as a continuous variable. As this variable was not normally distributed (Figure S1), it was examined using medians and interquartile ranges (IQR). Because age and education were significantly associated with the index score in univariate analyses (Table 2), quantile regressions, adjusted for age and education, were used to adjust for these potential confounders (Table 3). Significance for all analyses was set at *p* < 0.05.

**TABLE 2:** Distribution of the Torres Strait Healthy Ageing Index (TSHAI) score, in three categories (3–10), by participant characteristics.

Characteristics	Healthy ageing index score			Comparison	
	3–6	7–8	9–10		
	*n* (%)	*n* (%)	*n* (%)	Chi^2^	*p*
Total	42	91	116		
Demographics					
45–59 years	6 (14.3)	20 (22.0)	54 (46.6)	29.4	0.000
60–69 years	13 (31.0)	36 (39.6)	40 (34.5)		
70 years+	23 (54.8)	35 (38.5)	22 (19.0)		
Male	13 (31.0)	32 (35.2)	41 (35.3)	0.3	0.866
Female	29 (69.0)	59 (64.8)	75 (64.7)		
Single	24 (60.0)	53 (60.2)	49 (43.4)	6.8	0.034
Married/de facto	16 (40.0)	35 (39.8)	64 (56.6)		
Inner island	24 (57.1)	40 (44.0)	59 (50.9)	2.2	0.335
Outer island	18 (42.9)	51 (56.0)	57 (49.1)		
Highest education					
Primary	21 (56.8)	34 (37.8)	23 (20.0)	19.7	0.001
Any high school	7 (18.9)	26 (28.9)	38 (33.0)		
Postschool	9 (24.3)	30 (33.3)	54 (47.0)		
Employment type					
Unskilled	14 (42.4)	34 (44.7)	31 (36.5)	4.7	0.317
Semiskilled	7 (21.2)	26 (34.2)	26 (30.6)		
Skilled	12 (36.4)	16 (21.1)	28 (32.9)		
Languages					
English and/or Kriol	24 (57.1)	49 (53.8)	65 (57.0)	0.2	0.887
More languages	18 (42.9)	42 (46.2)	49 (43.0)		
Lifestyle					
No current alcohol	38 (90.5)	63 (69.2)	82 (70.7)	7.5	0.023
No previous alcohol	16 (38.1)	21 (23.1)	28 (24.1)	3.8	0.150
Not current smoker	35 (83.3)	77 (84.6)	99 (85.3)	0.1	0.952
Not previous smoker	11 (26.8)	26 (28.6)	36 (31.0)	0.3	0.857
Medical history					
No T2DM	9 (21.4)	36 (39.6)	46 (39.7)	5.0	0.083
No hypertension	16 (38.1)	22 (24.2)	49 (42.2)	7.5	0.023
No CKD	27 (64.3)	75 (82.4)	99 (85.3)	9.1	0.011
No stroke	32 (76.2)	89 (97.8)	114 (98.3)	31.5	0.000
No LOC	30 (78.9)	70 (79.5)	97 (84.3)	1.0	0.605
Vascular risk factors					
4+	23 (54.8)	33 (36.3)	33 (28.4)	13.8	0.008
2–3	15 (35.7)	45 (49.5)	52 (44.8)		
1–2	4 (9.5)	13 (14.3)	31 (26.7)		
Prescribing					
Polypharmacy	29 (72.5)	45 (52.3)	40 (38.8)	13.4	0.001
Overprescribing	16 (43.2)	38 (49.4)	40 (44.0)	0.6	0.736
Underprescribing	19 (50.0)	28 (37.3)	26 (28.3)	5.7	0.058
SOP	32 (76.2)	67 (77.9)	68 (66.7)	3.3	0.192
ACB	15 (39.5)	21 (28.0)	14 (15.2)	9.4	0.009

*Note:* Missing data excluded from denominators for the calculation of proportions. Number of vascular risk factors = sum of vascular risks (e.g., smoking, diabetes, hypertension, heart disease).

Abbreviations: ACB, Anticholinergic Cognitive Burden; CKD, chronic kidney disease; LOC, loss of consciousness; SOP, Suboptimal Prescribing; T2DM, type 2 diabetes mellitus.

**TABLE 3:** Distribution of median Torres Strait Healthy Ageing Index Score (TSHAI), by study variables.

Characteristic	Median (IQR)	*p*	*p*1	*p*2
Demographics				
45–59 years	9 (8–10)	<0.001**		
60–69 years	8 (7–9)			
70+ years	8 (6–9)			
Male (ref female)	8 (7–9)	0.508	0.428	0.457
Single	8 (7–9)	<0.05*	0.591	0.572
Married/de facto	9 (7–10)			
Inner island	8 (7–9)	0.883	0.533	0.331
Outer island	8 (7–9)			
Highest education				
Primary	8 (6–9)	<0.001**		
Any high school	9 (7–10)		0.194	
Postschool	9 (8–10)		0.071	
Employment type				
Unskilled	8 (7–9)	0.518		
Semiskilled	8 (7–9)		1.000	0.823
Skilled	8.5 (7–9.5)		0.099	0.396
Languages				
English and/or Kriol	8 (7–9)	0.848	1.000	0.876
More languages	8 (7–9)			
Lifestyle				
No current alcohol	8 (7–9)	0.092	0.441	0.677
No previous alcohol	8 (7–9)	0.157	0.582	0.611
Not current smoker	8 (7–9)	0.949	1.000	0.601
Not previous smoker	8 (7–10)	0.383	0.439	0.438
Medical history				
No T2DM	9 (7–10)	0.113	0.107	0.636
No hypertension	9 (7–10)	0.072	0.101	0.149
No CKD	8 (7–9)	<0.05*	0.201	0.807
No stroke	8 (7–9)	<0.001**	<0.001**	<0.001**
No LOC	8 (7–9)	0.280	0.133	0.115
Vascular risk factors				
4+	8 (6–9)	<0.05*		
2–3	8 (7–9)		0.272	0.054
1–2	9 (8–10)		<0.05*	<0.05*
Prescribing				
Polypharmacy	8 (7–9)	<0.05*	0.118	<0.05*
Overprescribing	8 (7–9)	0.681	0.837	0.646
Underprescribing	8 (6–9)	<0.05*	<0.05*	<0.05*
SOP	8 (7–9)	<0.05*	0.074	0.398
ACB	8 (6–9)	<0.05*	<0.05*	<0.05*

Abbreviations: ACB, anticholinergic cognitive burden; CKD, chronic kidney disease, LOC, loss of consciousness; SOP, suboptimal prescribing; T2DM, type 2 diabetes mellitus. *= *p* < 0.05; **= *p* < 0.001

The main analyses were repeated on a subset of participants aged ≥ 70 years to examine whether the trends observed were maintained for the eldest participants in the study (Tables S2 and S3). The data for participants who were aged ≥ 70 years and scored 9–10 on the index of healthy ageing were further analysed to provide a more complete description of this unique participant group.

Ethics approval was obtained from Queensland Health (HREC/13/QCH/129–878) and the James Cook University (H5495) Human Research Ethics Committee.

## 3 Results

There were 249 DPS participants with complete responses to all 10 measures of healthy ageing (Table S1). Figure 1 shows the distribution of healthy ageing index scores in three categories (scores of 3–6, 7–8 and 9–10) for three age groups (45–59, 60–69 and ≥ 70) for these 249 participants. Although younger age was significantly associated with higher healthy ageing index scores (Table 2), a high proportion of older adults (e.g., aged 70 and over) were living well and had high TSHAI scores (Figure 1). For example, among the 80 people aged 70 years and over, 35 (43.8%) had a TSHAI score of 7–8, and 22 (27.5%) had a score of 9–10.

Table 2 shows the distribution of the TSHAI score in three categories by participant characteristics. Significant factors in unadjusted analyses included marital status, education, lack of current alcohol use and absence of hypertension, chronic kidney disease and stroke. Higher scores were significantly associated with people having fewer vascular risk factors. The data also indicated the role of polypharmacy and prescribed anticholinergic load or burden [27]. After age adjustment using ordered probit regression, education, absence of stroke, number of vascular risk factors, polypharmacy, underprescribing and cholinergic load remained significant (results not tabled). The same trends were evident when the TSHAI was analysed as a continuous outcome and adjusted for age and education (Table 3).

When analyses were limited to participants 70 years and over (Table S2), and after age adjustment (Table S3), significant factors included inner island of residence (as distinct from outer island of residence), absence of type 2 diabetes mellitus, absence of stroke, a low number of vascular risk factors, polypharmacy, overprescribing, underprescribing and anticholinergic burden. The data for alcohol were not readily interpretable due to the low number of participants reporting consuming alcohol. In all adjusted analyses, the most consistent and outstanding factor was the absence of stroke.

## 4 Discussion

This study used data from a previously published dementia prevalence survey to explore factors associated with healthy ageing in the Torres Strait. The first step was the creation of the TSHAI. Subsequent analysis produced some expected findings and some unexpected outcomes. However, our results suggest that better access to education, a lower number of vascular risk factors and appropriate prescribing were significantly associated with healthy ageing in this cohort. Results were similar for older adults aged 70 years and over, except that living on an inner island was also protective. In all age groups, the absence of stroke was a strong predictor. This highlights the importance of stroke prevention strategies for this population [28].

The first step in this study was to develop a local healthy ageing index for the Torres Strait cohort based on local health issues and available data. This index has also yet to be formally validated against a ‘gold standard’ and is unlikely to gain that distinction any time soon. However, establishing face validity, if possible, will be examined shortly when the index is presented at the next Knowledge Circle meeting. Given the unique Torres Strait population and geographic background, the authors propose that it is still the best option available in this setting. None of the other published index‐based healthy ageing studies have included the 10 factors of this study or used them in this way. Few, if any, previous indices provide detailed analyses as to the relative merits of each indicator, especially when age adjusted.

The literature on healthy ageing in Australia's First Nation People provides little hard data although a comprehensive review of the key issues was published early in 2022 [2]. A culturally sensitive holistic approach to ageing must consider the colonial history and recognise the formidable hidden and obvious barriers Australia's First Nations People face in the ageing process. Several other publications have provided valuable qualitative information [29, 30] and proposed potential ways ahead. Coombes et al. (2018) suggested that each community has distinct strengths that support healthy ageing and programmes to support health ageing vary from one community to another. Furthermore, Yashadhana et al. (2022) highlighted that mainstream healthy ageing strategies are not appropriate for the First Nations population, who have a broader holistic view of ageing that includes connections to culture, Country and community.

In this Torres Strait study, the distribution of healthy ageing scores by age group (Figure 1) was predictable. However, some factors associated with healthy ageing seemed to change depending on the way the data were subsequently analysed. An unexpected finding was that hypertension, type 2 diabetes mellitus, chronic kidney disease and cigarette smoking were not shown to be more dominant indicators. It was not clear why this is the case for each factor, although when collectively considered as overall ‘vascular risk factors’, there does appear to be a negative correlation with healthy ageing. The age‐adjusted data for the cohort identified education, absence of a stroke and vascular risk factors as significant factors.

Polypharmacy was identified as a significant adverse factor for healthy ageing. The term ‘polypharmacy’ usually covers the number of different prescribed medications but without considering background conditions and medication indications. The published literature is not clear‐cut on these issues [31]. The reverse also appeared to be an important risk factor: lack of appropriate prescribing, which was a failure to prescribe medications such as antihypertensive agents, statins, aspirin and related drugs when clearly indicated. Of course, there are no specific data to support this assumption. Then there is ‘anti‐cholinergic burden’, where there is published evidence of accompanying adverse events, especially in people with cognitive impairment [32, 33].

For the participants 70 years and older, a newly identified factor was the island of residence. Over their lifetime, people from outer islands may have had less access to health and education services than those from the main administrative centre on Waiben. Type 2 diabetes mellitus also appeared to be a factor in this group. The relationship between type 2 diabetes mellitus and cognitive function in the Torres Strait has recently been explored [34]. But again, overall and in all analyses, the absence of stroke was the dominant factor for healthy ageing. This was not unexpected. Stroke can affect almost every aspect of daily function and in so many ways. This makes stroke prevention a public health priority [35].

The main strength of this study is that it is the first such investigation of healthy ageing conducted in Australia. Specifically, and to our knowledge, there are no other equivalent published reports, prospective or retrospective, of healthy ageing in Australia's First Nations People. This project also prompted the development of a local healthy ageing index based on function rather than surrogate markers. Even without formal validation, we believe this approach has considerable merit and could still be used, in its current or modified form, as part of subsequent prospective cohort studies of healthy ageing in regional, rural and remote settings.

The main limitation of the study was the retrospective use of data that was collected for another purpose. This approach potentially introduces bias and confounding. For example, there was no information on cardiac disease and, with regard to stroke, the presence or absence of atrial fibrillation nor information on hypertension control, cancer or dietary information. Finally, the sample was overrepresented by female participants. Thus, the results and conclusions must be viewed with some caution. The dataset was small, although of a size that could still be statistically analysed. This is another reason for caution. The number of variables collected in the study was also limited and primarily health focused and does not consider the broader socioeconomic and wider variables likely to impact on healthy ageing [2].

The main recommendation coming out of this study is that there needs to be a long‐term approach that involves well‐designed prospective cohort studies of ageing in this population. In the meantime, while stroke prevention takes priority, it is also important to address other key factors such as education, judicious prescribing and better management of type 2 diabetes mellitus. The challenge is there.

## Author Contributions

**Chenoa Wapau:** conceptualization, writing – original draft, methodology, writing – review and editing, formal analysis. **Malcolm McDonald:** writing – original draft, methodology, validation, writing – review and editing, supervision. **Fintan Thompson:** conceptualization, writing – original draft, methodology, validation, formal analysis, writing – review and editing, supervision. **Rachel Quigley:** writing – original draft, writing – review and editing. **Sarah G. Russell:** writing – original draft, writing – review and editing. **Betty Sagigi:** writing – original draft, writing – review and editing. **Gavin Miller:** writing – original draft, writing – review and editing. **Tania Korinihona:** writing – original draft, writing – review and editing. **Edward Strivens:** writing – original draft, writing – review and editing.

## Ethics Statement

Ethics approval was obtained from Queensland Health (HREC/13/QCH/129–878) and the James Cook University (H5495) Human Research Ethics Committee.

## Conflicts of Interest

The authors declare no conflicts of interest.

## Supporting information


Data S1.

## Data Availability

The data that support the findings of this study are sensitive. These data are not publicly available due to privacy and ethical restrictions. Data custodian approval and institutional approvals, such as ethics approval, would be required to enable the sharing of these data. Requests to access the datasets should be directed to the primary author.
