# Supplementary material for: What Makes for Healthy Ageing in the Torres Strait?
Source: Aust J Rural Health. 2025 Mar 7;33(2):e70020. doi: 10.1111/ajr.70020 (PMC11887416; doi:10.1111/ajr.70020)
Supplement: Supplementary file 1 — Data S1. [file AJR-33-0-s001.docx]

**Supplementary Table 1 – Distribution of responses for healthy ageing for 274 First Nations participants from the Dementia Prevalence Study in the Torres Strait region of Far North Queensland, Australia (2015-2018)**

| **Measure** |  | **Yes** | |  | **No** | |  | **Total** | |  | ***Missing*** |
| --- | --- | --- | --- | --- | --- | --- | --- | --- | --- | --- | --- |
|  |  | **n** | **(%)** |  | **n** | **(%)** |  | **n** | **(%)** |  | ***n*** |
| 1. Vision intact |  | 203 | (79.6) |  | 52 | (20.4) |  | **255** | (100.0) |  | *19* |
| 2. Hearing intact |  | 225 | (83.0) |  | 46 | (17.0) |  | **271** | (100.0) |  | *3* |
| 3. Mobility intact |  | 176 | (69.3) |  | 78 | (30.7) |  | **254** | (100.0) |  | *20* |
| 4. Minimal falls |  | 204 | (81.0) |  | 48 | (19.0) |  | **252** | (100.0) |  | *22* |
| 5. Continence intact |  | 195 | (76.8) |  | 59 | (23.2) |  | **254** | (100.0) |  | *20* |
| 6. Minimal pain |  | 230 | (90.6) |  | 24 | (9.4) |  | **254** | (100.0) |  | *20* |
| 7. No depression |  | 242 | (89.0) |  | 30 | (11.0) |  | **272** | (100.0) |  | *2* |
| 8. Cognition intact |  | 175 | (63.9) |  | 99 | (36.1) |  | **274** | (100.0) |  | *0* |
| 9. pALDs intact |  | 237 | (92.6) |  | 19 | (7.4) |  | **256** | (100.0) |  | *18* |
| 10. iADLs intact |  | 196 | (76.6) |  | 60 | (23.4) |  | **256** | (100.0) |  | *18* |
|  |  |  |  |  |  |  |  |  |  |  |  |
| **Complete responses** |  | **249** | **(90.9)** |  | **25** | **(9.1)** |  | **274** | **(100.0)** |  | *0* |

**Supplementary Table 2 – Distribution of the Torres Strait Healthy Ageing Index Score (TSHAI), in categories, by study variables, 70+**

|  | **Healthy Ageing Index Score** | | | | | |  | **Comparison** | |
| --- | --- | --- | --- | --- | --- | --- | --- | --- | --- |
| **Characteristics** | **3-6** | | **7-8** | | **9-10** | |  |  | |
|  | **n** | **(%)** | **n** | **(%)** | **n** | **(%)** |  | **Chi2** | **p** |
| **Total** | **23** |  | **35** |  | **22** |  |  |  |  |
| **Demographics** |  |  |  |  |  |  |  |  |  |
| Male | 8 | (34.8) | 13 | (37.1) | 9 | (40.9) |  | 0.2 | 0.912 |
|  |  |  |  |  |  |  |  |  |  |
| Single | 17 | (77.3) | 25 | (73.5) | 9 | (40.9) |  | 8.2 | 0.017 |
| Married/defacto | 5 | (22.7) | 9 | (26.5) | 13 | (59.1) |  |  |  |
|  |  |  |  |  |  |  |  |  |  |
| Inner island | 11 | (47.8) | 16 | (45.7) | 18 | (81.8) |  | 8.1 | 0.018 |
| Outer island | 12 | (52.2) | 19 | (54.3) | 4 | (18.2) |  |  |  |
| **Highest education** |  |  |  |  |  |  |  |  |  |
| Primary | 11 | (61.1) | 17 | (50.0) | 9 | (40.9) |  | 2.3 | 0.677 |
| Any high school | 3 | (16.7) | 10 | (29.4) | 6 | (27.3) |  |  |  |
| Post school | 4 | (22.2) | 7 | (20.6) | 7 | (31.8) |  |  |  |
| **Employment type** |  |  |  |  |  |  |  |  |  |
| Unskilled | 8 | (44.4) | 17 | (60.7) | 6 | (31.6) |  | 6.5 | 0.163 |
| Semi-skilled | 4 | (22.2) | 7 | (25.0) | 4 | (21.1) |  |  |  |
| Skilled | 6 | (33.3) | 4 | (14.3) | 9 | (47.4) |  |  |  |
| **Languages** |  |  |  |  |  |  |  |  |  |
| English and/or Kriol | 14 | (60.9) | 19 | (54.3) | 12 | (54.5) |  | 0.3 | 0.869 |
| More languages | 9 | (39.1) | 16 | (45.7) | 10 | (45.5) |  |  |  |
| **Lifestyle** |  |  |  |  |  |  |  |  |  |
| No current alcohol | 21 | (91.3) | 28 | (80.0) | 17 | (77.3) |  | 1.8 | 0.406 |
| No previous alcohol | 13 | (56.5) | 13 | (37.1) | 9 | (40.9) |  | 2.2 | 0.330 |
| Not current smoker | 19 | (82.6) | 30 | (85.7) | 18 | (81.8) |  | 0.2 | 0.913 |
| Not previous smoker | 8 | (36.4) | 14 | (40.0) | 7 | (31.8) |  | 0.4 | 0.823 |
| **Medical history** |  |  |  |  |  |  |  |  |  |
| No T2DM | 6 | (26.1) | 12 | (34.3) | 11 | (50.0) |  | 2.9 | 0.236 |
| No hypertension | 9 | (39.1) | 8 | (22.9) | 5 | (22.7) |  | 2.2 | 0.335 |
| No CKD | 13 | (56.5) | 27 | (77.1) | 19 | (86.4) |  | 5.5 | 0.063 |
| No stroke | 19 | (82.6) | 35 | (100.0) | 22 | (100.0) |  | 10.4 | 0.005 |
| No LOC | 15 | (75.0) | 30 | (88.2) | 18 | (81.8) |  | 1.6 | 0.454 |
| **Vascular risk factors** |  |  |  |  |  |  |  |  |  |
| 4+ | 11 | (47.8) | 12 | (34.3) | 6 | (27.3) |  | 2.3 | 0.678 |
| 2-3 | 9 | (39.1) | 18 | (51.4) | 13 | (59.1) |  |  |  |
| 1-2 | 3 | (13.0) | 5 | (14.3) | 3 | (13.6) |  |  |  |
| **Prescribing** |  |  |  |  |  |  |  |  |  |
| Polypharmacy | 15 | (68.2) | 19 | (55.9) | 9 | (42.9) |  | 2.8 | 0.247 |
| Over-prescribing | 8 | (38.1) | 18 | (56.3) | 7 | (46.7) |  | 1.7 | 0.427 |
| Under-prescribing | 9 | (42.9) | 10 | (32.3) | 5 | (33.3) |  | 0.7 | 0.718 |
| SOP | 16 | (69.6) | 27 | (79.4) | 13 | (68.4) |  | 1.0 | 0.592 |
| ACB | 10 | (47.6) | 9 | (29.0) | 4 | (26.7) |  | 2.4 | 0.298 |

Notes: T2DM=Type 2 Diabetes Mellitus, CKD=Chronic Kidney Disease, LOC=Loss of Consciousness, SOP=Suboptimal Prescribing, ACB=Anticholinergic Cognitive Burden. Missing data excluded from the denominators for calculation of proportions.

**Supplementary Table 3 – Distribution of the Torres Strait Healthy Ageing Index Score (TSHAI), by study variables, 70+**

| **Characteristic** | **Median (IQR)** | **p** |  | **p1** |
| --- | --- | --- | --- | --- |
| **Demographics** |  |  |  |  |
| Male (ref female) | 8 (6-9) | 0.353 |  | 0.086 |
| Single | 8 (6-8) | 0.09 |  | 1.000 |
| Married/defacto | 8 (7-9) |  |  |  |
|  |  |  |  |  |
| Inner island | 8 (7-9) | 0.086 |  | <0.05* |
| Outer island | 7 (6-8) |  |  |  |
|  |  |  |  |  |
| **Highest education** |  |  |  |  |
| Primary | 8 (6-8) | 0.47 |  |  |
| Any high school | 8 (7-9) |  |  | 1.000 |
| Post school | 8 (7-9) |  |  | 1.000 |
| **Employment type** |  |  |  |  |
| Unskilled | 7 (6-8) | 0.462 |  |  |
| Semi-skilled | 8 (6-9) |  |  | 0.196 |
| Skilled | 8 (6-9) |  |  | 0.163 |
| **Languages** |  |  |  |  |
| English and/or Kriol | 7 (6-9) | 0.344 |  | 0.066 |
| More languages | 8 (6-9) |  |  |  |
| **Lifestyle** |  |  |  |  |
| No current alcohol | 8 (6-9) | 0.260 |  | 1.000 |
| No previous alcohol | 7 (6-9) | 0.104 |  | <0.05* |
| Not current smoker | 8 (6-9) | 0.536 |  | 1.000 |
| Not previous smoker | 8 (6-8) | 0.737 |  | 1.000 |
| **Medical history** |  |  |  |  |
| No T2DM | 8 (7-9) | <0.05* |  | <0.05* |
| No hypertension | 8 (6-8) | 0.539 |  | 1.000 |
| No CKD | 8 (7-9) | <0.05* |  | 0.105 |
| No stroke | 8 (6.5-9) | <0.05* |  | <0.001** |
| No LOC | 8 (7-9) | 0.907 |  | 1.000 |
| **Vascular risk factors** |  |  |  |  |
| 4+ | 7 (6-8) | 0.17 |  |  |
| 2-3 | 8 (7-9) |  |  | <0.05* |
| 1-2 | 8 (6-9) |  |  | 0.172 |
| **Prescribing** |  |  |  |  |
| Polypharmacy | 7 (6-8) | 0.15 |  | 0.112 |
| Over-prescribing | 8 (7-8) | 0.454 |  | <0.05* |
| Under-prescribing | 7 (6-8) | 0.42 |  | 0.057 |
| SOP | 8 (6-8) | 0.69 |  | 1.000 |
| ACB | 7 (6-8) | 0.31 |  | <0.05* |

Notes: T2DM=Type 2 Diabetes Mellitus, CKD=Chronic Kidney Disease, LOC=Loss of Consciousness, SOP=Suboptimal Prescribing, ACB=Anticholinergic Cognitive Burden.


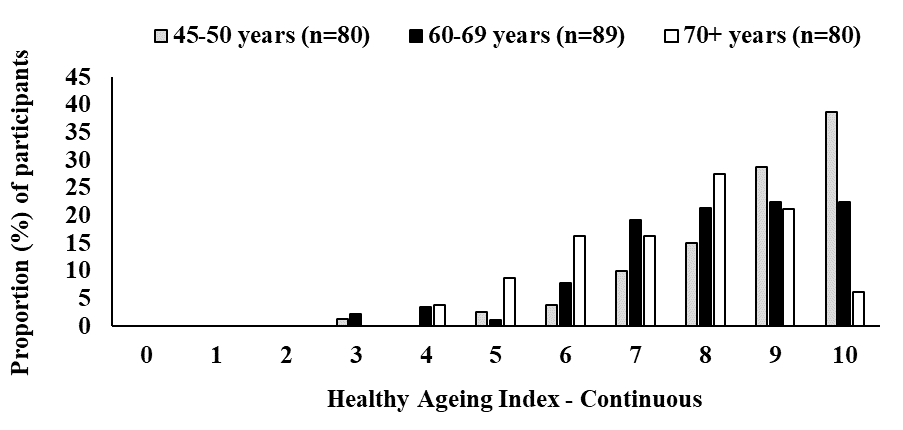


**Supplementary Figure 1 – Distribution of the Torres Strait Healthy Ageing Index (TSHAI) score as a continuous variable, by three age groups (45-59, 60-69, 70+), for 249 participants**
